# Simulation in Perioperative Liver Transplant Anesthesia: A Systematic Review

**DOI:** 10.7759/cureus.25602

**Published:** 2022-06-02

**Authors:** Thomas Oh, Ronit Patnaik, Jacob Buckner, Lucijana Krokar, Azan Ibrahim, Rehana S Lovely, Mustafa T Khan

**Affiliations:** 1 General Surgery, University of Texas (UT) Health San Antonio, San Antonio, USA; 2 Physiology and Anatomy, University of North Texas (UNT) Health Science Center, Fort Worth, USA

**Keywords:** medical residency, liver transplant anesthesiologist, education, surgery, peri-operative care, simulation, anesthesia, liver transplant

## Abstract

Due to the complexity of liver transplant patients and the variability in exposure to transplantation by anesthesia trainees, simulation is often required as an adjunct to clinical experience. This systematic review identifies current simulation models in the literature that pertain to perioperative liver transplant anesthesia.

Data were collected by performing an electronic search of the PubMed and Scopus databases for articles describing simulation in transplant anesthesia. Abstracts were screened using the preferred reporting items for systematic reviews and meta-analysis (PRISMA) guidelines. Three reviewers analyzed 16 abstracts found in the search and agreed upon articles that met the inclusion criteria for the systematic review.

A total of five publications met the inclusion criteria; they could be grouped as cognitive skills and technical skills simulators. Cognitive skills simulators utilized high-fidelity mannequins and animal models combined with traditional educational material to enhance pattern recognition of critical complications during liver transplantation. One manuscript focused on a technical skills acquisition by utilizing transesophageal echocardiography (TEE) to identify intraoperative pathologies.

There is a heterogeneity in the exposure to liver transplant care during anesthesia training. Simulation provides low-stakes exposure to the high-stakes skills required in the operating room. Hence, it can be used as an adjunct to improve both cognitive and technical skill acquisition for perioperative transplant anesthesia. The goal of these simulation programs is to improve patient outcomes and produce more capable anesthesiologists.

## Introduction and background

Anesthesiologists have been pioneers in developing and implementing simulation methodology in healthcare; however, these curriculums are not nationally standardized [1]. Anesthesia is sometimes described as “hours of boredom followed by moments of terror.” Simulation has been shown to improve patient outcomes and allows trainees to prepare for these critical moments by practicing high-stakes skills in a low-stakes environment [2-3]. Simulation is a core component of training in other professions involving high-stakes situations, such as aviation, emergency services, the military, and surgery. In 1999, the United States Army implemented simulation-based training as a core element for training combat medics, which led to a 10% drop in the pre-hospital mortality rate [4]. They reported that the increased use of simulation-based training allowed for faster skill acquisition, better retention, and standardization of medical skills among military personnel [4].

Anesthesiologists are crucial during the perioperative management of all surgical patients. The experience level of an anesthesiologist has a significant association with orthotopic liver transplant (OLT) outcomes [5]. Perioperative care of OLT patients is challenging for both the anesthesiologist and the surgical team. This procedure requires complex dissection and anastomoses prior to implantation of the healthy liver, leading to profound periods of hemodynamic instability, coagulopathy, electrolyte shifts, and metabolic derangements [6]. These physiologic instabilities can be demanding on an anesthetist’s skills. The anesthesiologists are expected to manage large volumes of blood transfusion, perform intraoperative critical care, communicate effectively with the surgical team, and display high levels of clinical acumen during these high-risk procedures. Due to these expectations, anesthesiologists typically complete a one- or two-year critical care or transplant fellowship.

To prepare for the potential perioperative challenges that arise while managing these complex and critically ill patients, anesthesia training programs have adopted simulation-based training. While multiple simulation training models are available, there is a lack of standardization in these curriculums [7]. To our knowledge, this is the first systematic review to focus on simulation in liver transplant anesthesia. Our review will serve as a reference for training programs interested in utilizing simulation to enhance perioperative management in liver transplantation.

## Review

Design and methods

Literature Search Strategy 

Manuscripts were compiled by scanning reference lists of articles manually and by performing an electronic search of the PubMed and Scopus databases for articles focusing on simulation in transplant anesthesia. Databases were searched from their inception to March 12, 2022. The Medical Subject Heading (MeSH) terms and keywords used to conduct this search can be found in Table 1.

**Table 1:** The Medical Subject Heading (MeSH) terms used for database search

Search#	Query	Results
1	(((("Kidney Transplantation") OR "Transplantation") OR "Heart Transplantation”) OR "Liver Transplantation") OR (Renal-transplant OR kidney transplant OR transplant OR cardiac-transplant OR heart-transplant OR liver-transplant OR "transplant surgery")	739,645
2	(("Anesthesia") OR ("Anesthesiologists")) OR (anesthesia OR anaesthesia)	346,324
3	#1 AND #2	5,462
4	(("Computer Simulation" OR "Patient Simulation" OR "Simulation Training")) OR (simulation OR surgical-simulation OR "virtual reality simulation" OR simulation-training)	445,921
5	#3 AND #4	16

**Figure 1:**
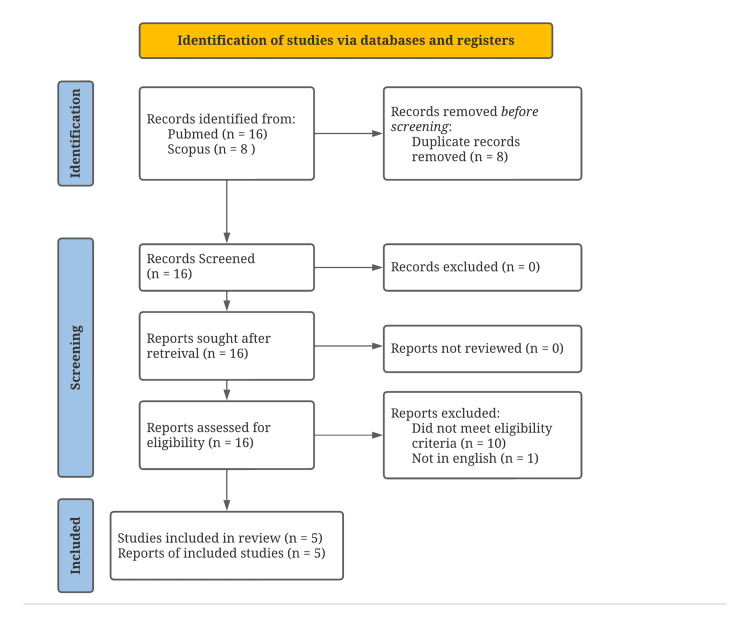
PRISMA 2020 flow diagram PRISMA: Preferred Reporting Items for Systematic Reviews and Meta-Analysis

Abstracts of the 16 articles found in the search were screened for relevance using the Preferred Reporting Items for Systematic Reviews and Meta-analysis (PRISMA) guidelines (Figure 1). Three reviewers analyzed all 16 abstracts and individually determined their eligibility. In cases of conflict, all reviewers discussed the merits of the article for this review and voted on inclusion or exclusion, leading to team consensus.

Inclusion and Exclusion Criteria

The inclusion criteria involved articles focusing on simulation in liver transplant anesthesia, simulation in perioperative care, education in interdisciplinary teams, and simulation in non-technical skills in transplant anesthesia. Simulation papers focused on other specialties of medicine (ie, critical care, surgery) as well as other subspecialties of anesthesia (ie, cardiothoracic anesthesia, critical care anesthesia) were excluded. 

Data Extracted

Each selected article was reviewed and the following data were obtained: the type of the study, aim of the study, sample size (n), type of subject (resident, advanced practice provider, attending physician), cost, type of skill being addressed (cognitive skill or technical skills), teaching modalities (didactics, mannequin simulation, live patient simulation, and other types of simulators used).

Results

Based on our inclusion criteria, five articles were included in this systematic review (Table 2). Four of the articles focused on cognitive skills and one focused on technical skills relevant to the perioperative care of liver transplant patients.

**Table 2:** Summary of publications in the systematic review TEE: Transesophageal echocardiography

Authors	Year	Type of skill	Modality	Summary
Aggarwal et al. [8]	2012	Cognitive	Didactics, mannequin, and live patient	Preoperative assessment of the patient, set-up of the operating room, and participation in intraoperative scenarios with a mannequin
Nguyen et al. [9]	2015	Cognitive	Mannequin	Assessment of resident's ability to manage intraoperative crises
Katz et al. [10]	2017	Cognitive	Virtual patient, serious gaming	Interactive perioperative management of virtual patients
Martin-Cancho et al. [11]	2011	Cognitive	Anesthetized porcine model	Management of hemodynamic stability, cardiac output, and electrolytes in a porcine model
Christensen et al. [12]	2021	Technical	Didactics, knowledge assessments, mannequin, and Vimedix TEE simulator	Curriculum to improve an anesthesiologist’s TEE knowledge and skill in liver transplant patients

Aggarwal et al. presented a simulation-based course that incorporated didactics, mannequin-based activities, and live patient training for OLT [8]. The curriculum involved 24 second and third-year anesthesia residents at a major academic center. The trainees started by performing a preoperative assessment of a patient with end-stage liver disease. Next, they set up the operating room and participated in various intraoperative scenarios with a mannequin. In these scenarios, trainees had to respond to various presentations such as hypotension and hyperkalemia. In the post-intervention surveys, participants unanimously reported that the simulation decreased anxiety, increased confidence, and increased awareness about the importance of communicating with the transplant team.

Nguyen et al. also reported on a mannequin-based transplant simulation experience in which they had anesthesia residents recognize and treat cardiac arrest during OLT reperfusion [9]. In this assessment, 25 residents were evaluated based on the time it took them to recognize the pathology and administer the treatment. Thirteen of these 25 subjects reported prior liver transplant experience. The authors showcased that residents with prior exposure to liver transplant anesthesia care were better equipped to recognize and treat the critically ill simulated patient in their scenario. They also suggest that simulation-based assessment of clinical skills is a useful tool for evaluating anesthesia resident readiness to address high-stress intraoperative situations. Post-simulation surveys of participants indicated they would unanimously recommend this simulation training to others.

Katz et al. described the development of a “serious game” that takes participants through interactive perioperative management of a virtual patient. The performance of each participant was assessed at the end of the game [10]. Serious games are interactive digital applications created for the purpose of imparting skills or knowledge that leverage the self-motivating elements of video games [13]. A total of 44 anesthesia residents were randomized to a “gaming group,” which had access to serious game training, and a control group that did not. Both groups had access to the same education materials outside of the gaming trainer and participated in a graded baseline simulation before reviewing the materials. After reviewing educational materials and literature, residents participated in a high-fidelity OLT patient simulation and were scored with a rubric developed using a modified Delphi method. While both groups experienced an increase in scores in all areas of the simulation, the gaming group displayed greater improvement than the control group in perioperative care.

Martin-Cancho et al. presented a series of porcine-based liver transplantation simulations. Their simulation experience was designed to improve the performance of anesthesia providers by demonstrating the physiologic changes that occur during orthotopic liver transplantation [11]. In this study, five trainees were evaluated based on their management of five anesthetized pigs who underwent vena-cava cross-clamping under total balanced anesthesia. The trainees were evaluated at different time points: at baseline, in the prehepatic phase, in the anhepatic phase, and in the neohepatic phase. Their performance was assessed based on their ability to maintain hemodynamic stability, cardiac output, electrolyte levels, and acid-base balance. With coaching during and between each iteration, all five participants showed substantial improvement in cognitive skills from their first simulation to their last.

Transesophageal echocardiography (TEE) is increasingly being used for intraoperative management during OLT. Christensen et al. described a course in which two board-certified cardiac anesthesiologists led 18 general anesthesiologists through a curriculum to improve liver transplant anesthesia TEE knowledge and skill [12]. The course included lecture and video-based didactic components as well as knowledge assessments. Participants learned the basics of TEE image acquisition, relevant anatomy, hemodynamic calculations, and pathology related to liver transplant patients. At the end of the simulation course, participants used the Vimedix TEE simulator (CAE Healthcare, Mainz, Germany) to assess various clinical scenarios and identify different pathologies. The authors reported that upon completing the curriculum, participants were able to identify the correct pathology. Furthermore, knowledge-based assessment scores improved from a median of 55% to 95%. Post-survey results indicated increased confidence in using TEE in perioperative care in liver transplant patients.

Discussion

Due to the variability in exposure to the management of liver transplant patients, anesthesia trainees would benefit from a standardized, efficient simulation curriculum to augment their training [14-15]. Our review examined five studies, detailing multiple ways in which simulation can augment education for anesthesia trainees. Four of the five studies targeted perioperative management while one focused specifically on using TEE during the intraoperative care of liver transplant patients.

Caring for patients undergoing liver transplantation is currently primarily based on direct experience at a relatively few institutions where the clinical volume is adequate to support training. While the number of transplant centers has increased in recent years, there is concern that this expansion decreased the average number of liver transplant cases available for anesthesia trainees [16]. Furthermore, due to the sporadic and limited exposure, anesthesiology training programs are not required by the Accreditation Council of Graduate Medical Education (ACGME) or the American Board of Anesthesiologists (ABA) to expose their residents to liver transplantation cases [17]. This has led to a very heterogenous experience between graduating anesthesiology residents across institutions.

To combat this dearth of exposure, various programs have incorporated simulation to create a more homogenous training experience across anesthesiology training programs [8]. The purpose of simulations is to hone both cognitive and technical skills relevant to the perioperative care of liver transplant patients. Hofer et al. reported that the quantity of anesthesia provider experience has a significant association with OLT outcomes [5]. In fact, they found that during the anesthesia provider’s first five cases, there was increased patient mortality and graft failure [5].

Cognitive skills required for intraoperative management of patients during OLT have traditionally been obtained through real-life clinical experiences. However, due to the uneven exposure to liver transplantation during training, not all trainees receive adequate exposure to obtain proficiency. In 2015, the 20 highest volume transplant centers averaged 136 liver transplants per year while the other 119 centers averaged only 37 [18]. Given this variability, only 26% of anesthesiology trainees reported participating in a structured transplant-related education [18]. Therefore, even high-volume centers may find it difficult to provide their residents with a standardized experience. Hence, simulation-based training can be utilized to enhance the development of cognitive and clinical skills relevant to transplant anesthesia.

Our systematic review revealed mannequin-based and porcine-based simulation activities designed to enhance the recognition and management of intraoperative complications such as hypotension and hyperkalemia. These experiential simulations provided reinforcement for pattern recognition of common critical complications that occur during liver transplantation. Furthermore, while transplant-specific simulations can improve the trainee’s aptitude in crisis management, general critical care skills, such as Advanced Cardiac Life Support (ACLS), should not be overlooked as adjuncts for skill improvement [19]. Novel simulators, such as the gaming tool included in this study, may also appeal more to new generations of physicians who are more comfortable with using technology [10]. Such methods may prove to be more efficient in the long run for anesthesia trainees versus traditional book learning.

During liver transplantation, patients undergo tremendous physiologic stress due to intraoperative hypotension and hemodynamic instability from fluid shifts and blood loss. Due to this, the technical skills of an anesthesiologist are of paramount importance. They must be comfortable with performing point-of-care ultrasounds, echocardiography, placing central lines, peripheral lines, and invasive hemodynamic monitors like arterial lines [20-23]. Although we did not find transplant anesthesia-specific training curriculums focusing on these skills, there are a plethora of part-task trainers in the literature that focus on improving these skills [20-23]. These trainers can be used to identify extra steps to increase speed and efficiency while decreasing complications. In our review, we found one part-task trainer that focused on enhancing the utilization of TEE to address intraoperative clinical scenarios [12]. Intraoperative use of TEE allows the anesthesiologist to monitor fluid status and diagnose pathologies such as pulmonary embolism, cardiac tamponade, heart failure, and air embolism.

An effective simulation-based curriculum for exposure to OLT would include a realistic representation of clinical events that challenge the trainee to learn the natural progression of liver transplantation [16]. Ideally, this curriculum would include objective metrics that educators could use to create a proficiency-based curriculum to ensure trainees reach proficiency benchmarks during their training [2]. This proficiency-based approach allows for uniform skill acquisition amongst a training cohort [24]. Furthermore, these simulation curriculums must showcase validity evidence using Messick’s validity framework, a proven method used in the education literature [25].

While this systematic review found a range of simulation models to improve perioperative anesthesia care for liver transplant patients, more work needs to be done. While the task trainers discussed above address some of the technical skills required by anesthesiologists managing liver transplant patients, they do not address the unique anatomy and physiology of end-stage liver disease patients. For this reason, there is a need to develop low-cost task trainer models designed to simulate the unique challenges posed by liver transplant patients [26-27]. Furthermore, while technical skills and medical management are crucial for improved patient outcomes, an aspect of simulation that should not be overlooked is multidisciplinary team training. Specifically, further work needs to be done to enhance skills such as having difficult conversations with families, interacting with organ procurement organizations, and working with surgical teams to improve quality control.

This review was not without limitations. Most of these publications did not report validity evidence and did not use Messick’s validity framework. More work needs to be done to develop structured curricula around these simulation models to maximize their use in anesthesia training. Furthermore, many of the isolated skills in transplant anesthesia are taught in general anesthesia or other subspecialties such as critical care and cardiac anesthesia; these studies were not specifically included. Finally, no studies in this paper addressed the cost of the simulators.

## Conclusions

The complex nature of liver transplant surgery can push the limits of even the most experienced anesthesiologists. The critical care needs of these ill patients can be both cognitively and technically challenging. Unfortunately, there is concern that the increasing number of institutions performing liver transplants around the United States has led to heterogeneity in exposure to transplant anesthesia for trainees. To combat this, training programs have begun using simulation as an adjunct to clinical experience. Simulation models allow trainees to practice in a low-stakes environment while managing the major complications that occur during high-stakes procedures. Hence, simulation can be used to improve cognitive and technical skill acquisition and better prepare anesthesia trainees for the perioperative management of transplant patients.
